# Testing a Sustainable Strategy Against Poultry Helminth Stages Developing in the Soil

**DOI:** 10.3390/pathogens14111168

**Published:** 2025-11-15

**Authors:** Jorge Alexander León, Gustavo Pérez-Anzúrez, Inês Abreu Ramos, Carlos Emiliano Magos Amado, David Boso Dafonte, João Lozano, José Ángel Hernández Malagón, Cristiana Cazapal-Monteiro, Rodrigo Bonilla, Jaime Sanchís, Adolfo Paz-Silva, Rita Sánchez-Andrade, Luís Manuel Madeira de Carvalho, María Sol Arias

**Affiliations:** 1 Control of Parasites Research Group (COPAR, GI-2120), Department of Animal Pathology, Faculty of Veterinary, University of Santiago de Compostela, 27002 Lugo, Spain; jorgealexander.leon@rai.usc.gal (J.A.L.); tavopzaz@gmail.com (G.P.-A.); inesabreu.ramos@usc.es (I.A.R.); al25510123@chapingo.mx (C.E.M.A.); david.boso@rai.usc.es (D.B.D.); joseangel.malagon@usc.es (J.Á.H.M.); cristiana.cazapal@usc.es (C.C.-M.); rodrigo.bonilla@carval.com.co (R.B.); sanchisjaime@gmail.com (J.S.); adolfo.paz@usc.es (A.P.-S.); rita.sanchez-andrade@usc.es (R.S.-A.); madeiradecarvalho@fmv.ulisboa.pt (L.M.M.d.C.); 2 Parasitic Diseases, Animal Sciences, University of Applied and Environmental Sciences (UDCA), Calle 222 #55-37, PBX (60 1) 6684700, Bogotá 111166, Colombia; 3 National Institute of Forestry, Agricultural and Livestock Research (INIFAP), National Center for Disciplinary Research in Animal Health and Safety (CENID-SAI), Jiutepec 62550, Morelos, Mexico; 4 CIISA—Centre of Interdisciplinary Research in Animal Health, Faculty of Veterinary Medicine, University of Lisbon, Avenida da Universidade Técnica, 1300-477 Lisbon, Portugal; jlozano@fmv.ulisboa.pt; 5 Associate Laboratory for Animal and Veterinary Sciences (AL4AnimalS), Avenida da Universidade Técnica, 1300-477 Lisbon, Portugal; 6 CARVAL Pharmaceuticals, Calle 15 # 32-450 Km 2 Vía Acopi, Yumbo 760502, Colombia; 7 Parasitology and Parasitic Diseases, University of La República—CENUR Litoral Norte, Salto 50000, Uruguay

**Keywords:** prevention, parasites, birds, free-range, parasitophagous fungi, spraying

## Abstract

Free-ranging hens are at risk of infection by parasites characterized by certain stages that develop in the soil until attaining the infective phase. To analyze the usefulness of a biological control strategy of helminths affecting pasturing hens, fecal samples containing eggs of the helminths *Ascaridia galli* and *Capillaria* spp. were collected and then homogenized with an electric mixer. A total of 64 small areas were established by placing wooden frames (15 × 40 × 30 cm) on the ground and then adding approximately 100 g of a fecal mixture (per area). Four batches of 16 areas were considered: G1, sprayed with 2 × 10^6^ spores of the parasitophagous fungus *Mucor circinelloides* (day 0) at 0.5 L/m^2^ (=600 mL/area); G2, sprayed with spores twice (every two weeks); G3, sprayed four times (every week); and Control, sprayed weekly with water. After a four-week period, the egg viability reduced for ascarids and capillarids (26% and 27%, respectively) in the control group; 64% and 79% in G1; 71% and 82% in G2; and 79% and 80% in G3. It was concluded that spraying with fungal spores provides a very useful tool for preventing infection by helminths on free-range poultry.

## 1. Introduction

Over the past two decades, significant changes have been introduced to improve the welfare of poultry [1]. One of the main indications relies on ensuring that laying hens have enough space available by using enriched cages, or through alternative systems also called non-cage systems containing nest boxes, litter, perforated platforms, and sometimes elevated perches [2]. While 445 cm^2^ per white-egg-laying hen in cages has been counseled in the USA [3], this value is higher in the European Union (EU) (750 cm^2^) to prevent major welfare issues [4]. Besides this, it has been pointed out by consumers that high stocking densities negatively impact hens’ natural behaviors [5,6].

When hens are maintained under outdoor management and enjoy pastures, strong stimuli for exploratory behavior can be received, improving the possibilities of dust-bathing or scratching the ground looking for bugs, earthworms, or slugs. Free-range systems ensure access to the outdoors when weather conditions are appropriate, mainly during daytime [7], but the risk of infection by certain parasites developing different stages (eggs, cysts, larvae) in the ground can also be enhanced [8]. The common life cycle of certain helminths involves infected individuals passing their eggs in feces, and once in the soil and under proper conditions (temperature and humidity mainly), some changes occur, with the final result of their infective stage being reached, which is most commonly characterized by the presence of a larval stage inside [9]. The occurrence of the nematode *Ascaridia galli* has dramatically increased within the past decade in commercial laying hens on farms in some European countries after the implementation of the EU directive 1999/74/EC establishing minimum standards for the protection of laying hens [10]. The importance of infection increases due to their ability to transport other pathogens, as occurs with *A. galli* for *Salmonella* spp., or with *Heterakis gallinarum* for *Histomonas meleagridis* [7].

Since the control of these endoparasites is mainly performed using anthelmintics, a significant increase in frequency of deworming in laying flocks has been recorded in several EU countries, and only benzimidazole drugs (e.g., flubendazole and fenbendazole) have been approved for treatment of *A. galli* (larval and adult stages) within the EU member states [11]. An attempt to develop an effective deworming control strategy implied that the accumulation of parasite eggs in a chicken coop could be prevented through early detection of infection, followed by repeated anthelmintic treatments at strategic times. This should lead to a significant reduction in parasite burden and egg excretion at the flock level, although more frequent treatments should be needed [12]. The emergence of parasites resistant to certain drugs, such as ivermectin, associated with their excessive use and/or insufficient dosage, exacerbates the situation [13]. It should be noted that, although the use of parasiticides was initially limited only to situations in organic production systems where the lives of animals were at risk, economic and environmental concerns, as well as the detection of chemical residues in animal-derived products, have led to the imposition of significant restrictions [14].

The use of agents for the biological control of parasites that develop their infective stages in the soil and affect animals and even humans in some cases has been proposed [15,16,17,18]. Among the possibilities described, parasiticide fungi have been gaining importance. These are filamentous saprophytic species that under normal conditions feed on decaying organic matter, feces, lignin, and other sources, and have developed the ability to also feed on the propagation stages of some parasites (oocysts, eggs, larvae, etc.). Regarding the targeted stages, those parasitophagous fungi are elementarily classified into ovicide *(Mucor circinelloides*, *Pochonia chlamydosporia*, *Purpureocillium lilacinum*, *Trichoderma atrobrunneum),* larvicide *(Duddingtonia flagrans*, *Monacrosporium thaumasium, Arthrobotrys* spp.)*,* and entomopathogenic *(Metarhizium* spp., *Beauveria bassiana*) [19,20,21,22].

In order to provide a sustainable and environmentally friendly strategy to prevent infection by certain helminths, an experimental trial was designed based on spraying spores from *Mucor circinelloides* a saprophytic filamentous fungus with ovicidal and coccidicidal activity, directly onto fecal samples collected from grazing laying hens and placed in small areas of soil, in order to mimic the field conditions in which these parasites evolve until they reach the infective stage.

## 2. Materials and Methods

### 2.1. Parasitophagous Fungi

The filamentous saprophytic fungus *Mucor circinelloides* (Spanish Type Culture Collection-CECT-20824) was isolated from feces collected from cattle and horse farms in Galicia (NW Spain), and then it was cultured in a submerged medium until a concentration of > 10^6^ chlamydospores/mL was reached. This is a fungal species with demonstrated antagonism on protozoan oocysts, and eggs of trematodes and nematodes (roundworms, whipworms) [23]. The medium is composed of 7.1 g NaCl, 1.6 g Na_2_HPO_4_·12 H_2_O (Sigma-Aldrich, Madrid, Spain), and 30.6 g wheat (*Triticum aestivum*) per L of water.

### 2.2. Collection and Analysis of Fecal Samples

Twelve samples of feces from free-range laying hens were collected from a private poultry farm in Galicia, and after their analysis using the McMaster flotation test, the presence of eggs of the helminths *A. galli* and *Capillaria* spp. was detected. Consequently, fecal samples were collected again for a week and then homogenized with an electric mixer.

The protocol of the McMaster flotation test consisted of weighing three grams of feces and dissolving them in 42 mL of water. After filtering the blend through a 150 μm sieve, the fecal dilution was recovered into two 15 mL tubes and centrifuged for 10 min at 1500 rpm [24]. Once the supernatant was discarded, saturated saline solution was added (specific gravity 1.2), the total content was homogenized again, and after waiting for a 5 min period, a McMaster chamber (the two cells) was filled and observed under an optical microscope at 100–200× total magnifications. The results obtained were indicated as the number of eggs of parasites per gram of feces (EPG), by using a multiplication factor of 50.

### 2.3. Design of the Study

**Figure 1:**
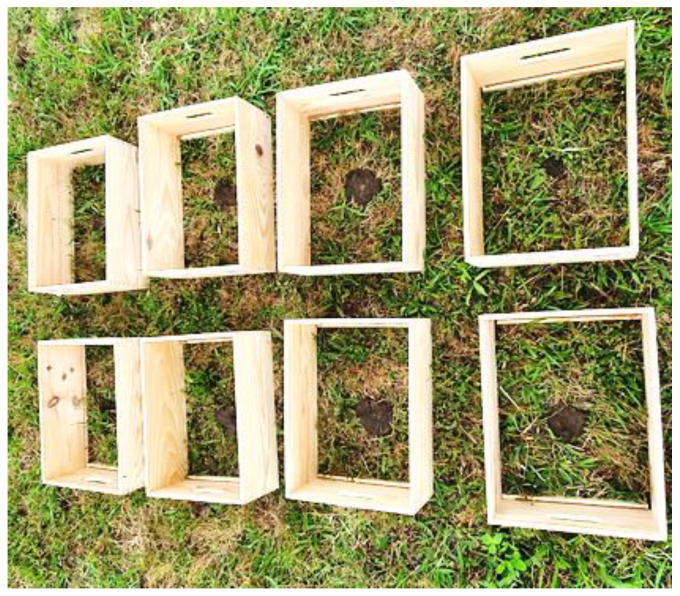
Small areas were delimited in a grassland with the aim of checking the effect of *M. circinelloides* on eggs of gastrointestinal nematodes passed in feces from free-range laying hens (original photo).

In order to test the antagonism of *M. circinelloides* on the eggs of gastrointestinal nematodes that affect free-ranging hens, a grassy meadow was used where hens or any other grazing animal species had never been kept. A total of 64 small areas were marked out on the ground using wooden frames (15 × 40 × 30 cm), and then approximately 100 g of the fecal mixture was added to the center of the space delimited by each of them (Figure 1).

**Figure 2:**
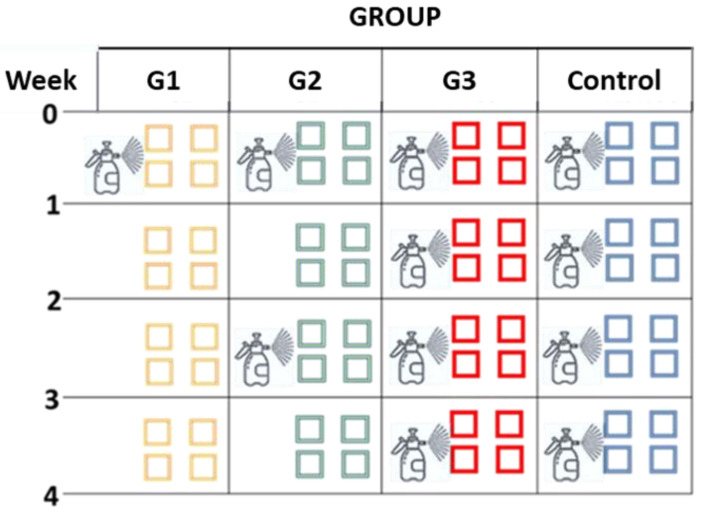
A total of four groups were considered in the current research: G1 was sprayed once with chlamydospores of *M. circinelloides*, G2 twice, and G3 four times, while the Group Control was sprayed four times with water (original scheme).

As represented in Figure 2, four groups were considered in this study: Group G1 was sprayed once with 2 × 10^6^ chlamydospores of *M. circinelloides* (day 0) at 0.5 L/m^2^ (=600 mL/area); G2, sprayed with spores twice (every two weeks); G3, sprayed four times (every week); and Control, sprayed weekly with water.

### 2.4. Evaluation of Parasiticidal Activity in Soil

During a period of four weeks, soil samples were collected weekly from four frames in each group, to a depth of 5 cm, and taken to the Zoonosis and Public Health Laboratory (Faculty of Veterinary Medicine, University of Santiago de Compostela, Lugo, Spain). The samples were always collected from the center of the frames using a garden trowel (5 cm wide × 10 cm long), since, as indicated above, feces were always placed in this location. The trowel was cleaned with soap and water between each sample collection and then dried with paper.

The analysis consisted of emulsifying the soil in water (at a rate of 100 mL of 5% Tween 20 per 100 g), stirring until a homogeneous mixture was obtained, and leaving it to stand for 12 h. Then, after passing it successively through 300, 150, 60, and 38 µm filters, the solution was washed with water for 30 min. The sediment retained in the 38 µm mesh was transferred to a 1 L sedimentation cup, distilled water was added to 2/3 of the surface, and it was left to stand again for 12 h. Once this time had elapsed, the supernatant was discarded, and the sediment was placed in 12 mL tubes (occupying 1/4), and the rest was filled with water. After gently shaking to resuspend the pellet, 20 µL aliquots were taken from each tube and placed between a glass slide and a cover slip for microscopic examination of ≥100 parasite eggs to check for alterations, viability, and developmental status, as further described.

### 2.5. Morphological Characterization of Helminth Eggs During the Trial

**Figure 3:**
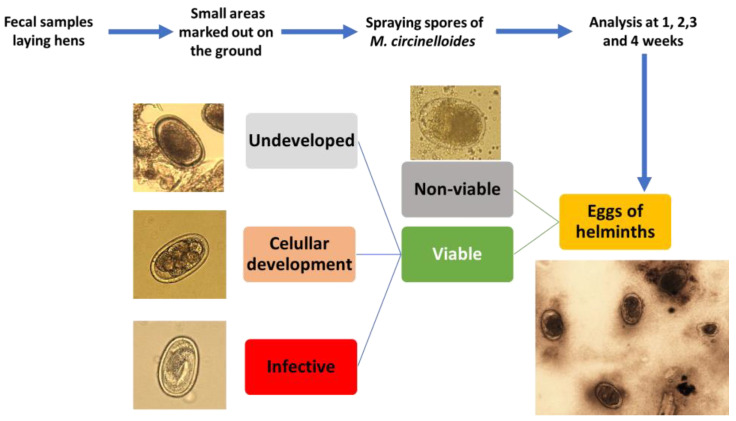
The evaluation of the effect of *M. circinelloides* involved the recovery of helminth eggs and their observation under an optical microscope to classify them regarding their viability and stage of development (original photos and scheme).

Based on microscopic observation, the eggs of helminths were first classified as non-viable and viable, depending on the integrity of the eggshell, and possible alterations in the cytoplasm (vacuolized cytoplasm, asymmetrical embryonic development, destruction) [25]. The viable eggs were then further grouped according to their stage of development into viable undeveloped eggs (zygote), viable in cellular development (two or more cells), and viable infective (containing a larva inside) (Figure 3).

Data obtained by the examination of the eggs was used to calculate the percentages of Viability Reduction (VR) and Infection Risk (IR), according to the following formulas:

Viability Reduction: VR (%) = [1 − (Non-viable forms/Viable forms)] × 100

Infection Risk: IR (%) = (Infective forms/Viable forms) × 100

### 2.6. Statistical Analysis

The statistical package IBM^®^ SPPS^®^ Statistics v.22 (IBM Corporation, Armonk, NY, USA) was used in the current investigation to analyze the number of eggs in the different groups, by taking into account their viability and developmental stage. Since these counts did not adjust to a normal distribution, the nonparametric Kruskal–Wallis test was used, and differences were considered significant if *p <* 0.05.

## 3. Results

### 3.1. Parasitological Findings

The analysis of fecal samples taken from free-range laying hens showed the presence of eggs of *A. galli* (264–326 EPG) and *Capillaria* spp. (237–311 EPG). Oocysts of protozoan species were also observed.

### 3.2. Antagonistic Effect by Spraying Spores of M. circinelloides

#### 3.2.1. On the Viability of Eggs of Helminths

At the beginning of the trial (week 0), the viability of eggs of *A. galli* was 91 ± 4%, and 84 ± 7% in eggs of *Capillaria* spp.

**Figure 4:**
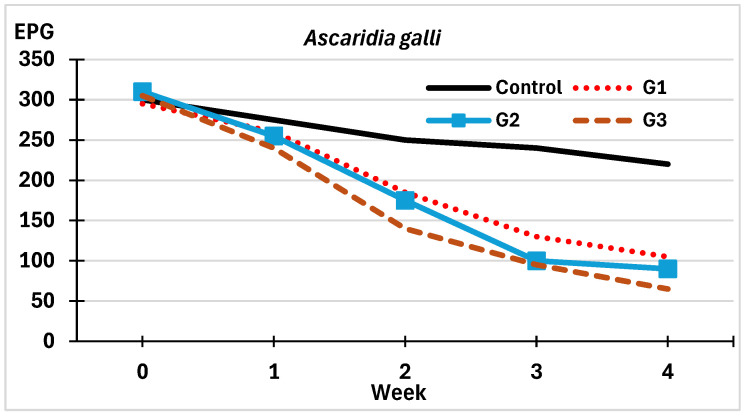
Variations on the counts of viable eggs of *A. galli* in feces from free-range laying hens. G1: sprayed once with chlamydospores of *M. circinelloides* (red-dotted line); G2: twice (blue line); G3: four times (brown-dashed line); Control: sprayed four times with water (black line).

As shown in Figure 4, the dynamics of viable eggs of *A. galli* in the control group revealed a slight to moderate decrease over time, with a reduction of approximately one quarter between the initial and final burden values.

In the groups sprayed with *M. circinelloides* spores, the number of viable eggs decreased from the second week onwards, with values at the end of the trial (4th week) significantly lower than the initial values (64.4% in G1, 71% in G2, and 78.7% in G3). These differences were statistically significant between the Control and the groups exposed to *M. circinelloides* (χ^2^ = 15.333, *p *= 0.012), but not among the three fungus-treated groups (χ^2^ = 0.954, *p *= 0.845).

The estimation of VR values showed that the presence of viable eggs of *A. galli* decreased steadily in the control group from the first week onwards (Table 1). In G1, a significant reduction of 37.3% was recorded two weeks after exposure to *M. circinelloides*, which increased rapidly until the end of the trial. An analogous pattern was obtained in G2 and G3, although higher VR values were recorded. Significant differences were observed between the control group and the three treated groups, and between G1 and G2 and G3 (χ^2^ = 20.814, *p *= 0.009, and χ^2^ = 6.402, *p *= 0.040, respectively).

**Figure 5:**
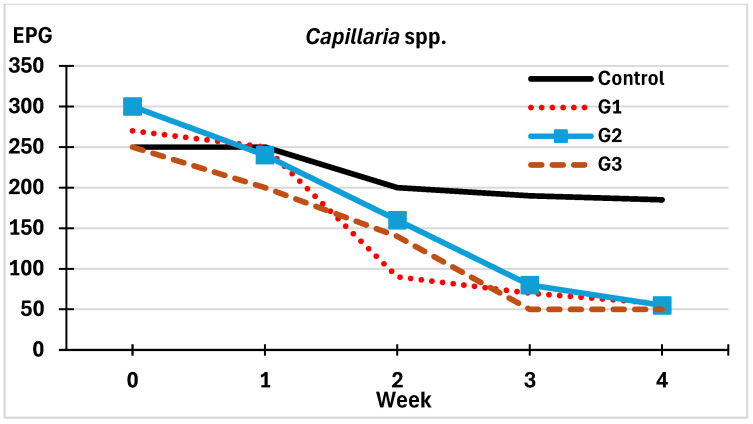
Variations on the counts of viable eggs of *Capillaria* spp. in feces from free-range laying hens. G1: sprayed once with chlamydospores of *M. circinelloides* (red-dotted line); G2: twice (blue line); G3: four times (brown-dashed line); Control: sprayed four times with water (black line).

Findings concerning the numbers of viable eggs of *Capillaria* spp. are shown in Figure 5. Similar values were recorded in all groups during the first week, with variations of around a quarter observed in the control group, while in the groups sprayed with chlamydospores of *M. circinelloides*, differences of close to 80% were detected between the first and fourth weeks (Figure 5).

When calculating the VR (Table 2), it was demonstrated that the viability of eggs of *Capillaria* spp. in the group Control decreased steadily after the second week. In groups exposed to *M. circinelloides*, the egg viability was reduced by 44% or more from the second week onwards, with percentages of around 80% recorded after four weeks. Significant differences were obtained between the Control and the groups sprayed with the fungal spores (χ^2^ = 7.104, *p *= 0.029). The highest VR values were recorded in G2, but significant differences were not found among the three treated groups (χ^2^ = 0.883, *p *= 0.902).

#### 3.2.2. On the Development of Eggs of Helminths

**Figure 6:**
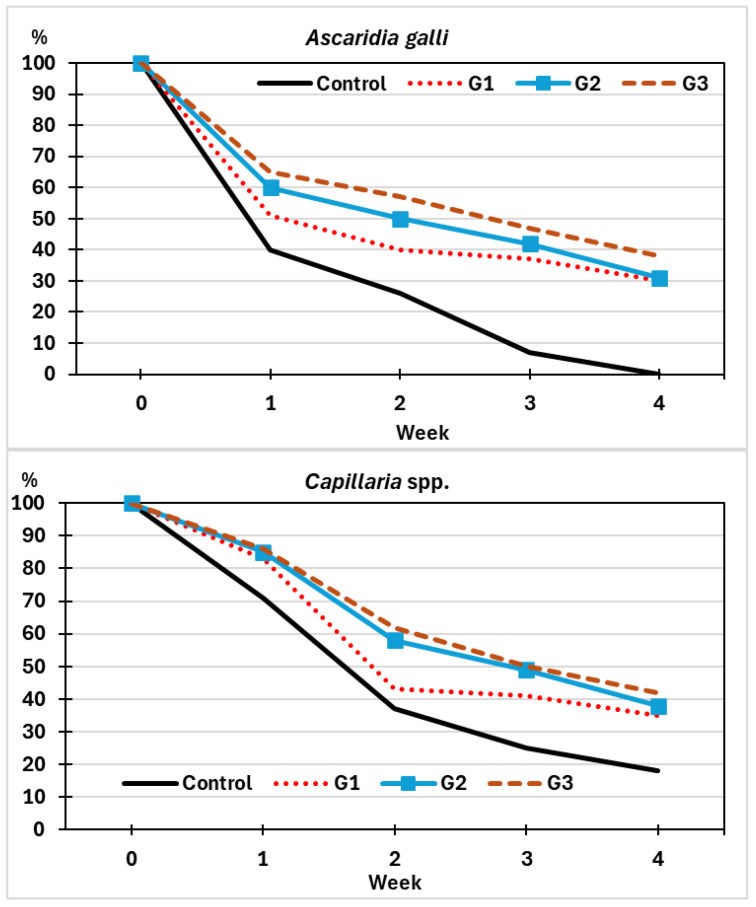
Kinetics of eggs of helminths identified as undeveloped (*A. galli*—first chart; *Capillaria* spp.—second chart). G1: sprayed once with chlamydospores of *M. circinelloides* (red-dotted line); G2: twice (blue line); G3: four times (brown-dashed line). Control: sprayed four times with water (black line).

In the group Control, the number of undeveloped (viable) eggs decreased rapidly throughout the trial (Figure 6), and after one week (*A. galli*) or two weeks (*Capillaria* spp.), more than half of the eggs showed cellular development (Figure 6). At the end of the trial, no undeveloped eggs of *A. galli* were observed, while 18% of the eggs of *Capillaria* spp. remained without development at the same time.

Regarding the groups exposed to *M. circinelloides*, a slower development was recorded for both helminth eggs, especially in G2 and G3, and nearly 40% of the eggs of *A. galli* and *Capillaria* spp. did not develop after a four-week interval. Significant differences were demonstrated between the Control group and those exposed to the fungus (χ^2^ = 7.098, *p *= 0.009).

#### 3.2.3. On the Infection Risk by Helminths

**Figure 7:**
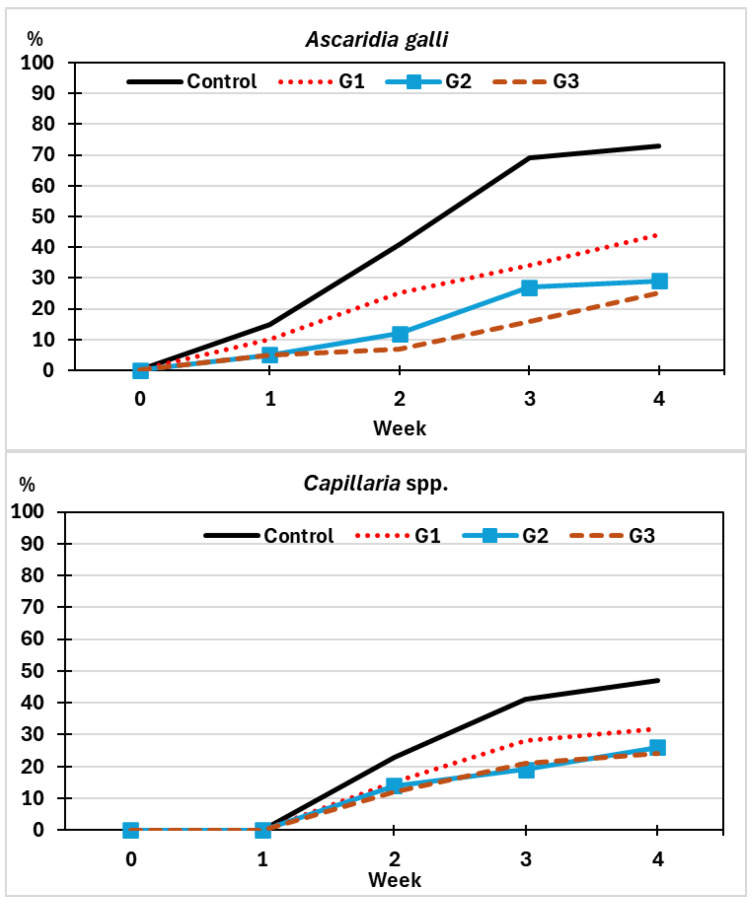
Variations on the values of Infection Risk (IR) for each helminth species (*A. galli*—first chart; *Capillaria* spp.—second chart). G1: sprayed once with chlamydospores of *M. circinelloides* (red-dotted line); G2: twice (blue line); G3: four times (brown-dashed line); Control: sprayed four times with water (black line).

The presence of infective eggs of *A. galli* (containing a L3—third stage larva—inside was recorded in the first week in the Control group and increased significantly until the fourth week, reaching an infection risk (IR) value of 73% (Figure 7). With respect to *Capillaria* spp., infective eggs were observed from the second week, achieving an IR of 47% at the end of the assay.

In the groups sprayed with *M. circinelloides* chlamydospores, the counts of infected eggs of *A. galli* increased gradually throughout the test, although less than the group Control, and in the fourth week reached IR percentages of 44% in G1, 29% in G2, and 25% in G3 for *A. galli*. Concerning *Capillaria* spp., the pattern was similar, and the IR values were lower than 30% at the end of the trial.

Significant differences were demonstrated between the Control group and those receiving the chlamydospores of *M. circinelloides* (χ^2^ = 9.054, *p* = 0.013). Although no differences were observed among the three groups treated with the parasitophagous fungus (χ^2^ = 2.771, *p* = 0.355), the lowest IR values were achieved in G3 for *A. galli*, and in G2 and G3 for *Capillaria* spp.

## 4. Discussion

Controlling parasites that affect animals with frequent access to pastures remains a difficult and pending task, despite the availability of effective drugs with parasiticidal activity registered for veterinary use. This poses a serious problem when promoting free-range poultry farming [26]. For the purpose of searching for a viable and sustainable tool to contribute to reducing the risk of infection by helminths, a strategy involving the distribution of a saprophytic filamentous fungus, *Mucor circinelloides*, was conducted. The protocol consisted of spraying the fungal chlamydospores directly on fecal samples from free-range laying hens infected by *A. galli* and *Capillaria* spp., which resulted in a significant reduction in the viability of the eggs of the aforementioned helminths. These results agree partly with a previous assay performed with the fungus *Pochonia chlamydosporia* [16], as well as it has been reported that the antagonistic effect of *M. circinelloides* against oocysts of *Eimeria* spp. affecting free-range peacocks [24].

Infection of laying hens by *A. galli* nematodes is responsible for poor body condition, reduced welfare, and decreased egg production [27]. Birds infected by species belonging to the genus *Capillaria* can show nonspecific signs, namely diarrhea, poor body condition, weight loss, emaciation, slow growth, reduced egg production, and even death. Most efforts against them have been focused on deworming, and successful results have been achieved by the administration of flubendazole in drinking water for 7 days, although birds can become infected again within one week after deworming [11], because the infective stages in the soil remained unaffected by the benzimidazole. Since these two nematodes are transmitted directly via the fecal-oral route by eggs containing a larva inside (L3 in *A. galli,* and L1 in *Capillaria* spp.), it appears quite conceivable that current welfare-friendly cage-free housing systems might increase the risk of infection [28]. In the current study, the administration of chlamydospores of *M. circinelloides* was assayed during a four-week period following three different frequencies (once, twice and four times), and the viability of eggs of *A. galli* dropped by two-thirds, and by more than three-quarters in eggs of *Capillaria* spp. These results corroborate prior trials encompassing the spraying of *M. circinelloides* on eggs of the pig roundworm (*Ascaris suum*) shed in feces from piglets [29]; similarly, the viability of eggs of *Trichuris* spp. excreted in feces of captive dromedaries decreased by up to one-third [30]. It is important to note that the number of eggs of *Capillaria* spp. found throughout the study could be underestimated, since the use of sieves with a mesh size of 38 µm could allow some of these eggs to pass through, although the counts detected were significant, and it was possible to evaluate the antagonistic effect of *M circinelloides* on them.

An important issue related to the eggs of certain helminths, such as *Ascaridia* spp. and *Capillaria* spp., resides in their ability to survive in range soil and remain viable for two years or more [16]. Under optimal environmental conditions, mainly humidity, temperature and vegetation (darkness/sunlight protection), cellular development triggers inside the eggs until an infective larval stage is attained [31,32]. Given that infection occurs after ingesting infectious stages through eating contaminated soil, food, or water, it seems that a practical approach is to prevent or avoid the development of eggs in soil and/or feces. In the present research, it has been demonstrated that *M. circinelloides* is able to delay or stop the development of eggs of *A. galli* and *Capillaria* spp. in feces from laying hens, as evidenced by the observation that nearly 80% of the eggs of both parasites remained undeveloped after four weeks of exposure, while 80–100% of the eggs in the Control group (without fungi) did develop throughout the study. It should be stressed that ingesting undeveloped eggs does not imply any risk of infection.

As mentioned above, some changes have been introduced in the egg-laying poultry sector with a view to improving the welfare of the birds, mainly by providing them with more space and opportunities to interact with their environment through the possibility of foraging, sunbathing, and running [33]. Consequently, systems such as organic/free-range egg production have experienced a notable boost because they comply with new legal requirements and meet consumer demands. However, these regimes have been linked to a higher risk of infection among hens [34]. In Sweden, 77% of free-range/organic birds were found to be infected with roundworms [35], which is consistent with a subsequent study involving organic birds from eight European countries [36]. Studies conducted on free-range laying hens under mountain farming conditions (Northern Italy) showed that almost all hens were parasitized by at least one helminth species [26]. Based on the demonstration in the present research that significant reductions concerning the risk of infection were achieved by spraying feces containing helminth eggs with chlamydospores of *M. circinelloides*, it is highly conceivable that this could be an effective, feasible, and eco-friendly approach to try to limit the level of contamination by these pathogens in the soil and, therefore, the risk of infection among pasturing hens.

Organic and free-range production systems must provide areas for free grazing for birds, but these areas also provide suitable conditions for the life cycle of certain helminths, which may increase the likelihood of infection by these pathogens [32]. Some hypotheses have been argued with the aim to explain the detection of high levels of parasite infection among free-range/organic hens, which comprise contact with feces of wild birds, the presence of earthworms as intermediate hosts (for *H. gallinarum* and some *Capillaria* species), and the cumulative contamination proceeding from previous flocks, then after an initial entry, successive flocks of hens might infect each other [37]. Besides this, soil contamination by wildlife species should also be taken into consideration [34].

Use of several species of saprophytic filamentous fungi has provided highly successful results in the sustainable control of some parasites affecting domestic and sylvatic mammals, especially among those maintained under semi-extensive regimes with free access to grasslands [19,38,39,40]. One of the most important concerns relates to the method of delivery, and different formulations containing parasiticidal fungal spores have been tested, including commercially manufactured nutritional pellets, direct spraying onto feed before ingestion, and edible gelatins [24,41,42,43]. In the present study, a strategy was tested on feces of free-ranging hens containing eggs of two helminths, *A. galli* and *Capillaria* spp., and consisted of spraying with chlamydospores of *M. circinelloides* during a four-week period, once, twice, and four times. The best results in terms of reducing egg viability and egg development to infective stages were obtained by spreading the fungus every seven (four times) to fifteen days (twice). The absence of differences based on the frequency of application leads us to believe that it seems more appropriate to do so every two weeks to make the task easier for farmers, although, to state this more definitely, it would be advisable to extend the trial for a longer period.

Poultry products are considered the most widely consumed source of animal protein by humans, and their production is subject to strict legislative regulations [44]. Free-ranging hens can contribute to improvement of soil chemical properties and soil fertility [45]. Moreover, organic eggs have been demonstrated to be the most distinguishable, especially in terms of yolk color and nutritional composition [46]. The importance of infections by helminths affecting hens has led to the search for feasible and efficient solutions. It is important to consider that only benzimidazoles are allowed for poultry in the USA and the EU, are frequently administered when the flocks present high levels of infection, and that it may be needed to repeat the deworming several times during a production cycle [47,48]. The restrictions that apply for deworming laying hens include compliance with the withdrawal periods to prevent drug residues in eggs; thus, deworming the entire flock to avoid reinfection is indicated. This solution is difficult and not very practical for free-range birds. Moreover, anthelmintic resistance must also be taken into consideration [49]. A strategy utilizing targeted anthelmintic treatment and enhanced diagnostic methods has recently been proposed [28]. Despite these solutions, the finding that hens in a contaminated pasture can become reinfected within a week of the end of treatment reinforces the idea that deworming is a temporary solution, and little or no effect on parasites developing in soil is achieved [47,50]. For instance, it has been assumed that nine out of ten eggs of *A. galli* present in feces are capable of completing their development after one or two weeks under optimal laboratory conditions, a period that is longer under field conditions [38]. Although many ascarid eggs are destroyed within a few months, a small proportion (up to 3%) can survive for up to two years, and thus the level of soil contamination by infective eggs increases steadily [16,51]. In consequence, avoiding the accumulation of parasite stages in soil appears to be a helpful approach to prevent further infections and thus the need for deworming.

## 5. Conclusions

Parasites such as roundworms (*Ascaridia* spp.) or threadworms (*Capillaria* spp.) are known as soil-transmitted helminths because their transmission to animals and even humans occurs after the ingestion of infective eggs developing in soil. Accordingly, any attempt to control these infections on free-ranging hens should include beneficial actions to restrain or delay their evolution until the respective infective stages. By delimiting small areas on the ground using wooden frames, a very practical and reliable procedure is available for analyzing the antagonistic effect of *M. circinelloides* on helminths that reach the soil through feces and develop in the environment, i.e., under field conditions. In the current study, after two weeks of exposure to chlamydospores of *M. circinelloides*, both the viability and infection risk of eggs of *A. galli* or *Capillaria* spp. decreased by half or more when compared to the control group. Several studies have been designed and implemented to confirm these results among flocks of hens maintained under free-range regimes.

## Figures and Tables

**Table 1:** Values of Viability Reduction (% VR) of eggs of *A. galli* in feces from free-range laying hens.

Group	Week			
	1	2	3	4
**Control**	8.3	16.7	20.0	26.7
**G1**	11.9	37.3	55.9	64.4
**G2**	17.7	43.5	67.7	71.0
**G3**	21.3	54.1	68.9	78.7

G1: sprayed once with chlamydospores of *M. circinelloides*; G2: twice; G3: four times. Control: sprayed four times with water.

**Table 2:** Values of Viability Reduction (VR) of eggs of *Capillaria* spp. in feces from free-range laying hens.

Group	Week			
	1	2	3	4
** Control **	0.0	20.0	24.0	26.0
** G1 **	7.4	66.7	74.1	78.9
** G2 **	20.0	46.7	73.3	81.7
** G3 **	20.0	44.0	80.0	80.0

G1: sprayed once with chlamydospores of *M. circinelloides*; G2: twice; G3: four times. Control: sprayed four times with water.

## Data Availability

The data sets used are available from the corresponding author upon reasonable request.

## References

[1] Alig B.N., Anderson K.E., Malheiros D.M., Harding K.L., Malheiros R.D. (2025). Assessment of the Effects of Stocking Density on Laying Hens Raised in Colony Cages: Part II—Egg Production, Egg Quality, and Welfare Parameters. Poultry.

[2] Hahn D.L. (2014). The Effects of Feed Additives, Housing Systems, and Stress on *Salmonella* Shedding in Single Comb White and Brown Laying Hens. Ph.D. Dissertation.

[3] United Egg Producers (2017). Animal Husbandry Guidelines for U.S. Egg-Laying Flocks..

[4] European Union (1999). Council Directive 1999/74/EC of 19 July 1999 Laying Down Minimum Standards for the Protection of Laying Hens. Off. J. Eur. Communities.

[5] Mench J.A., Blatchford R.A. (2014). Determination of Space Use by Laying Hens Using Kinematic Analysis. Poult. Sci..

[6] Riddle E.R., Ali A.B.A., Campbell D.L.M., Siegford J.M. (2018). Space Use by 4 Strains of Laying Hens to Perch, Wing Flap, Dust Bathe, Stand and Lie Down. PLoS ONE.

[7] Knierim U. (2006). Animal welfare aspects of outdoor runs for laying hens: A review. NJAS Wagening. J. Life Sci..

[8] Pillay R., Naidoo P., Duma Z., Bhengu K.N., Mpaka-Mbatha M.N., Nembe-Mafa N., Mkhize-Kwitshana Z.L. (2024). Potential Interactions Between Soil-Transmitted Helminths and Herpes Simplex Virus Type II: Implications for Sexual and Reproductive Health in Sub-Saharan African. Biology.

[9] Schlosser-Brandenburg J., Midha A., Mugo R.M., Ndombi E.M., Gachara G., Njomo D., Rausch S., Hartmann S. (2023). Infection with soil-transmitted helminths and their impact on coinfections. Front. parasitol..

[10] Thapa S., Thamsborg S.M., Wang R., Meyling N.V., Dalgaard T.S., Petersen H.H., Mejer H. (2018). Effect of the nematophagous fungus *Pochonia chlamydosporia* on soil content of ascarid eggs and infection levels in exposed hens. Parasites Vectors.

[11] Tarbiat B., Jansson D.S., Tydén E., Höglund J. (2016). Comparison between anthelmintic treatment strategies against *Ascaridia galli* in commercial laying hens. Vet. Parasitol..

[12] Tarbiat B., Jansson D.S., Höglund J. (2022). Implementation of a targeted treatment strategy for the sustainable control of *Ascaridia galli* infections in laying hens. Vet. Rec. Open.

[13] Sulik M., Antoszczak M., Huczyński A., Steverding D. (2023). Antiparasitic activity of ivermectin: Four decades of research into a “wonder drug”. Eur. J. Med. Chem..

[14] Jackson F., Miller J. (2006). Alternative approaches to control--quo vadit?. Vet. Parasitol..

[15] Thamsborg S.M., Roepstorff A., Larsen M. (1999). Integrated and biological control of parasites in organic and conventional production systems. Vet. Parasitol..

[16] Thapa S., Thamsborg S.M., Meyling N.V., Dhakal S., Mejer H. (2017). Survival and development of chicken ascarid eggs in temperate pastures. Parasitology.

[17] Valadão M.C., Millena de Carvalho L., Vieira Í.S., Neves P.H., Ferreira V.M., Campos A.K., Elias de Freitas Soares F., Ferraz C.M., Ribeiro Vilela V.L., Braga F.R. (2020). Germination capacity of the *Pochonia chlamydosporia* fungus after its passage through the gastrointestinal tract of domestic chickens (*Gallus gallus domesticus*). Exp. Parasitol..

[18] Felici M., Tugnoli B., Ghiselli F., Baldo D., Ratti C., Piva A., Grilli E. (2023). Investigating the effects of essential oils and pure botanical compounds against *Eimeria tenella* in vitro. Poult. Sci..

[19] Bilotto F., Fusé L.A., Sagües M.F., Iglesias L.E., Fernández A.S., Zegbi S., Guerrero I., Saumell C.A. (2018). Predatory effect of *Duddingtonia flagrans* on infective larvae of gastro-intestinal parasites under sunny and shaded conditions. Exp. Parasitol..

[20] Canhão-Dias M., Paz-Silva A., Madeira de Carvalho L.M. (2020). The efficacy of predatory fungi on the control of gastrointestinal parasites in domestic and wild animals-A systematic review. Vet. Parasitol..

[21] Mendoza-de Gives P., López-Arellano M.E., Olmedo-Juárez A., Higuera-Pierdrahita R.I., von Son-de Fernex E. (2023). Recent Advances in the Control of Endoparasites in Ruminants from a Sustainable Perspective. Pathogens.

[22] Lozano J., Cunha E., de Carvalho L.M., Paz-Silva A., Oliveira M. (2024). First insights on the susceptibility of native coccidicidal fungi *Mucor circinelloides* and *Mucor lusitanicus* to different avian antiparasitic drugs. BMC Vet. Res..

[23] Hernández J.Á., Sánchez-Andrade R., Cazapal-Monteiro C.F., Arroyo F.L., Sanchís J.M., Paz-Silva A., Arias M.S. (2018). A combined effort to avoid strongyle infection in horses in an oceanic climate region: Rotational grazing and parasiticidal fungi. Parasites Vectors.

[24] Lozano J., Almeida C., Vicente E., Sebastião D., Palomero A.M., Cazapal-Monteiro C., Arias M.S., Oliveira M., Carvalho L.M., Paz-Silva A. (2024). Assessing the efficacy of the ovicidal fungus *Mucor circinelloides* in reducing coccidia parasitism in peacocks. Sci. Rep..

[25] Cruz L.M., Allanson M., Kwa B., Azizan A., Izurieta R. (2012). Morphological changes of *Ascaris* spp. eggs during their development outside the host. J. Parasitol..

[26] Wuthijaree K., Lambertz C., Gauly M. (2017). Prevalence of gastrointestinal helminth infections in free-range laying hens under mountain farming production conditions. Br. Poult. Sci..

[27] Sharma N., Hunt P.W., Hine B.C., Ruhnke I. (2019). The impacts of *Ascaridia galli* on performance, health, and immune responses of laying hens: New insights into an old problem. Poult. Sci..

[28] Höglund J., Daş G., Tarbiat B., Geldhof P., Jansson D.S., Gauly M. (2023). *Ascaridia galli*—An old problem that requires new solutions. Int. J. Parasitol. Drugs Drug Resist..

[29] Cortiñas F.J., Cazapal-Monteiro C.F., Hernández J.A., Arroyo F.L., Miguélez S., Suárez J., López de Arellano M.E., Sánchez-Andrade R., Mendoza de Gives P., Paz-Silva A. (2015). Potential Use of *Mucor circinelloides* for the Biological Control of Certain Helminths Affecting Livestock Reared in a Care Farm. Biocontrol Sci. Technol..

[30] Hernández J.A., Cazapal-Monteiro C.F., Arroyo F.L., Silva M.I., Palomero A.M., Paz-Silva A., Sánchez-Andrade R., Arias M.S. (2018). Biological Control of Soil Transmitted Helminths (STHs) in a Zoological Park by Using Saprophytic Fungi. Biol. Control.

[31] Anane A., Dufailu O.A., Addy F. (2022). *Ascaridia galli* and *Heterakis gallinarum* prevalence and genetic variance of *A. galli* in rural chicken from the Northern Region, Ghana. Vet. Parasitol. Reg. Stud. Rep..

[32] Wongrak K., Daş G., Moors E., Sohnrey B., Gauly M. (2014). Establishment of gastro-intestinal helminth infections in free-range chickens: A longitudinal on farm study. Berl. Münch. Tierarztl. Wochenschr..

[33] Bestman M., Verwer C., van Niekerk T., Leenstra F., Reuvekamp B., Amsler-Kepalaite Z., Maurer V. (2019). Factors related to free-range use in commercial laying hens. Appl. Anim. Behav. Sci..

[34] Bestman M., van Niekerk T., Göransson L., Ferrante V., Gunnarsson S., Grilli G., Arndt S.S., Bas Rodenburg T. (2023). Free-range use and intestinal parasites in organic/free-range laying hens. J. Appl. Poult. Res..

[35] Jansson D.S., Nyman A., Vågsholm I., Christensson D., Göransson M., Fossum O., Höglund J. (2010). Ascarid infections in laying hens kept in different housing systems. Avian Pathol..

[36] Thapa S., Hinrichsen L.K., Brenninkmeyer C., Gunnarsson S., Heerkens J.L., Verwer C., Niebuhr K., Willett A., Grilli G., Thamsborg S.M. (2015). Prevalence and magnitude of helminth infections in organic laying hens (*Gallus gallus domesticus*) across Europe. Vet. Parasitol..

[37] Walker J.G., Morgan E.R. (2014). Generalists at the interface: Nematode transmission between wild and domestic ungulates. Int. J. Parasitol. Parasites Wildl..

[38] Faedo M., Larsen M., Grønvold J. (2002). Predacious activity of *Duddingtonia flagrans* within the cattle faecal pat. J. Helminthol..

[39] Paoletti B., Iorio R., Morelli S., Di Teodoro L., De Angelis E., Bartolini R., Di Cesare A. (2024). A Pilot Study of the in Vitro Efficacy of Different Concentrations of *Duddingtonia flagrans* for the Control of Gastrointestinal Nematodes of Sheep. Ann. Parasitol..

[40] Mendoza-de Gives P., López-Arellano M.E., Aguilar-Marcelino L., Olazarán-Jenkins S., Reyes-Guerrero D., Ramírez-Vargas G., Vega-Murillo V.E. (2018). The nematophagous fungus *Duddingtonia flagrans* reduces the gastrointestinal parasitic nematode larvae population in faeces of orally treated calves maintained under tropical conditions-Dose/response assessment. Vet. Parasitol..

[41] Voinot M., Cazapal-Monteiro C., Hernández J.Á., Palomero A.M., Arroyo F.L., Sanchís J., Pedreira J., Sánchez-Andrade R., Paz-Silva A., Arias M.S. (2020). Integrating the control of helminths in dairy cattle: Deworming, rotational grazing and nutritional pellets with parasiticide fungi. Vet. Parasitol..

[42] Paz-Silva A., Salmo R., Viña C., Miguel Palomero A., Ángel Hernández J., Sánchez-Andrade R., Cazapal-Monteiro C., Sol Arias M. (2023). Gelatin Treats Containing Filamentous Fungi to Promote Sustainable Control of Helminths among Pets and Zoo Animals. Biol. Control.

[43] Salmo R., Viña C., Zubiria I., Malagón J.Á.H., Sanchís J.M., Cazapal C., Arias M.S., Sánchez-Andrade R., Paz-Silva A. (2024). Formulating Parasiticidal Fungi in Dried Edible Gelatins to Reduce the Risk of Infection by *Trichuris* sp. among Continuous Grazing Bison. Pathogens.

[44] Rahimian S., Gauly M., Daş G. (2016). Embryonation ability of *Ascaridia galli* eggs isolated from worm uteri or host faeces. Vet. Parasitol..

[45] Soares P.R., Pato R.L., Dias S., Santos D. (2022). Effects of Grazing Indigenous Laying Hens on Soil Properties: Benefits and Challenges to Achieving Soil Fertility. Sustainability.

[46] Dalle Zotte A., Cullere M., Pellattiero E., Sartori A., Marangon A., Bondesan V. (2021). Is the farming method (cage, barn, organic) a relevant factor for marketed egg quality traits?. Livest. Sci..

[47] Tarbiat B., Enweji N., Jansson D.S., Wallström E., Osterman-Lind E., Höglund J. (2023). A follow-up on the Swedish roundworm control program: Strengths and weaknesses. JAPR.

[48] Feyera T., Sharpe B., Elliott T., Shifaw A.Y., Ruhnke I., Walkden-Brown S.W. (2022). Anthelmintic efficacy evaluation against different developmental stages of *Ascaridia galli* following individual or group administration in artificially trickle-infected chickens. Vet. Parasitol..

[49] Zirintunda G., Biryomumaisho S., Kasozi K.I., Batiha G.E., Kateregga J., Vudriko P., Nalule S., Olila D., Kajoba M., Matama K. (2022). Emerging Anthelmintic Resistance in Poultry: Can Ethnopharmacological Approaches Offer a Solution?. Front. pharmacol..

[50] Tarbiat B., Jansson D.S., Höglund J. (2015). Environmental tolerance of free-living stages of the poultry roundworm *Ascaridia galli*. Vet. Parasitol..

[51] Höglund J., Jansson D.S. (2011). Infection dynamics of *Ascaridia galli* in non-caged laying hens. Vet. Parasitol..

